# Do it yourself: discerning the effects of self-directed activity on conceptual learning

**DOI:** 10.1038/s41539-025-00364-9

**Published:** 2025-09-29

**Authors:** Garvin Brod, Elfriede Holstein, Leonie Weindorf, Joseph Colantonio, Elizabeth Bonawitz, Maria Theobald

**Affiliations:** 1 DIPF | Leibniz Institute for Research and Information in Education, and Center for Individual Development and Adaptive Education of Children at Risk (IDeA), Frankfurt, Germany; 2 Department of Psychology, Goethe University, Frankfurt, Germany; 3 Harvard University, Cambridge, USA; 4 Department of Psychology, University of Trier, Trier, Germany

**Keywords:** Psychology, Human behaviour

## Abstract

Can you learn better by doing something yourself (DIY) or by watching somebody else do it? We present a new approach to examine this perennial question in research on learning and instruction. In a science learning task, children aged 5 to 7 years (*N* = 95) either generated predictions themselves (active condition) or observed the predictions of a fictitious other child (yoked condition) before seeing the outcome. Unlike previous yoked designs, we first emulated responses from a Bayesian learner. Critically, these responses were then individually matched to each yoked child given their unique prior beliefs at the start of the experiment. This novel approach allowed us to discern the effects of DIY on conceptual learning much more clearly than before. We found that actively generating predictions led to deeper conceptual understanding than observing another’s matched predictions, and that this advantage of DIY was associated with an increased experience of agency.

## Introduction

Many foundational theories of human learning argue that learning is an active construction process and that it is therefore beneficial to actively involve students in constructing knowledge, most notably by letting them perform knowledge-generating activities themselves^1–5^. There are many arguments in favor of this “do it yourself” (DIY) approach. In cognitive psychology terms, DIY may lead to a more thorough evaluation of the problem structure and of how the observed data relates to your prior knowledge, leading to more elaborative encoding processes^6^. An alternative, non-mutually exclusive, account could be that DIY leads to an experience of agency, which increases intrinsic motivation to learn. Consistent with this explanation, giving participants agency over decisions—even if they are sham decisions—has been shown to upregulate the mesolimbic dopamine system, which modulates memory formation in the hippocampus^7^. There are thus clear psychological mechanisms that suggest that DIY is conducive to learning.

On the other hand, from a simple rational perspective, it makes no difference whether you perform an action yourself or just observe someone else—as long as the other person tests your hypotheses (or more informative hypotheses), the informativeness of the evidence remains the same^8–10^. Moreover, various research suggests that observing knowledgeable others, such as teachers, can be particularly effective for learning causal structures^11^. This is because knowledgeable others can point learners to just the data and statistical relations that are most helpful for causal inference^12,13^. Consistent with these findings from basic research, classroom studies suggest that children often benefit more from explicit instruction than from hands-on scientific activities, such as conducting their own experiments^14,15^ (but see ref. ^16^). However, in cases where explicit instruction outperforms self-directed activity, this advantage may stem from learners receiving more relevant or targeted evidence. A more rigorous test of the effect of instructional mode would require controlling the informational content across conditions to ensure that both groups receive equivalent evidence. In sum, it is currently unclear whether and, if so, which aspects of DIY are conducive to learning.

How can we discern the effects of DIY on learning? We chose to focus on a very simple experimental contrast: making predictions yourself vs. observing the predictions of a fictitious other learner. This manipulation builds on prior research in children’s causal learning, which often compares a group that actively performs actions with a control group that passively observes a real or fictitious individual^17–20^. Such *yoked* control conditions are ideal for investigating the effects of self-directed activity, as they ensure that learners in both conditions are exposed to the same data for the same amount of time^6^. However, our experimental manipulation is different from previous yoking manipulations in cognitive and developmental psychology, where participants could influence the course of events by selecting the content or sequence of an intervention^17,20,21^. In contrast, children in our study could only predict the outcomes of pre-determined interventions. This design allowed us to hold the informativeness of the evidence constant across conditions and eliminated potential confounds related to children’s ability to design informative experiments^22^. In sum, comparing active and yoked predictions promises to reveal the most fundamental aspect of DIY: the ability to perform a volitional action, even if this action does not alter the course of events.

However, the comparison of active and yoked predictions can still lead to an unfair bias in favor of the active condition. For example, consider what happens if you are in the yoked group and the person you are yoked to predicts something you would not predict. You will likely be confused and preoccupied with thoughts that are not helping your learning, such as why the other learner is predicting this. You might also become frustrated, which further reduces your motivation to engage with the task. This is likely to happen frequently since different individuals bring different prior knowledge and beliefs to a task. To really discern whether doing something yourself has an advantage over watching somebody else do it, this other person needs to do the same thing that you would have done. Therefore, it would be ideal to yoke participants to themselves, i.e., to present the same actions that the participant in the yoked condition would have made had they been in the active condition.

The issue of fit to the yoked learner may be particularly important. Evidence suggests that children do not readily extend inferences from a taught other to themselves if they do not perceive the other as similar. For example, children who observed another child being taught later behaved as if they themselves were being taught directly, whereas children who observed an adult being taught did not^23^. This finding aligns with broader research showing that children learn effectively from peers—through imitation^24–27^, peer informants^28^, and peer interaction^29–31^. Taken together, these results show the children are savvy peer learners, sensitive to whether observed peer learning is relevant to them, and rationally deploy inferences in indirect social learning contexts.

The goal of the current study was twofold. First, to provide a proof of concept that such a personalized yoking procedure is possible–at least to a certain extent–using a novel Bayesian yoking approach. Second, to test the hypothesis that DIY remains beneficial for learning even under this new yoked control condition. Unlike previous research on causal learning with yoked controls, this design ensured that participants could not choose the evidence they encountered, keeping the available evidence identical across conditions. This hypothesis was based on literature suggesting that the experience of agency that can be assumed to go along with DIY is conducive to children’s learning^19,32^. In sum, this new approach would allow us to determine the core effect of agency on causal learning much more clearly than previous approaches.

We tested children aged 5–7 on a prediction–feedback task in which they had to learn about what determines the amount of water displacement. We focused on this age group because most children at this age have misconceptions about water displacement^33,34^, making it an ideal scenario for investigating how they revise their intuitive causal models. Moreover, children in this age group are generally capable of explicit belief revision when confronted with counterevidence^35^, allowing us to investigate the factors that support such change. Children in the yoked control condition saw the predictions of a (fictitious) other child, which were in fact the responses of an ideal Bayesian learner personalized to have that participant’s individual prior beliefs (i.e., Bayesian yoking procedure). The Bayesian approach is particularly suitable here because learners are faced with a problem of inference under uncertainty when learning from testing predictions (i.e., they do not know what the true theory is). By specifying learners’ prior theories and their degrees of belief in these theories, Bayes’ rule allows this problem to be solved optimally according to probability theory. Recent work has shown that children’s choices, and thus their belief revision during this task, closely resemble those of an optimal Bayesian learner tuned to each child’s prior knowledge^36^. Therefore, the Bayesian learner will mostly respond as the participants in the yoked condition would have responded had they been in the active condition. At worst, the Bayesian learner learns a little faster, but as research on observing knowledgeable others shows^11,23^, this could also be beneficial for the participants.

## Results

### Accuracy of Bayesian Yoking Procedure

The yoking procedure involved “binning” children in the yoked condition to one of six predefined profiles that best matched the individual child’s prior beliefs. The dominant belief per profile and the number of children assigned to each of the six profiles were as follows: Profile 1: unclear/random belief (*n* = 4); Profile 2: predominant mass belief (*n* = 8); Profile 3: predominant material belief (*n* = 18); Profile 4: predominant size belief (*n* = 5); Profile 5: unclear/random belief (*n* = 11); Profile 6: mass with predominant size belief (*n* = 0). This distribution confirms that, consistent with previous research, most children began the task with misconceptions focused on material or mass. While this binning approach was necessary for practical reasons, pilot testing and simulations indicated that it effectively captured children’s priors with high precision. Nonetheless, one might be concerned that in our actual data, the cluster (and resulting observed yoked outcomes) that each individual child was assigned would not be an ideal match. To assess the accuracy of our Bayesian Yoking Procedure, we tested how well it approximated each child’s optimal learning path. On average, yoked responses matched an individualized ideal model 94.6% of the time (range: 79–100%), with 43% of children (20/46) receiving a perfect match. Importantly, children with a perfect match and children with a slight mismatch did not differ in performance in the posttest (79% vs. 83%, *t*(44) = −0.48, *p* = 0.632) or transfer test (92% vs. 88%, *t*(44) = 0.47, *p* = 0.638). These results indicate that the yoking procedure provided a highly accurate match to an ideal learning trajectory, with no observable impact from slight mismatches.

While qualitatively exploring the agreement between the optimal Bayesian responses and the children’s actual responses, we noticed that most of the differences stem from children learning at a slightly lower rate than the optimal Bayesian learner and/or flipping back to their previous theory occasionally which the Bayesian learner never does. These results confirm that the yoked profiles we employed closely matched individualized models, and replicate our previous modeling findings. Thus, these results support the validity of our Bayesian yoking procedure, as they indicate that children in the yoked condition see the responses that they would have given themselves or slightly better ones.

### Learning performance

**Fig. 1:**
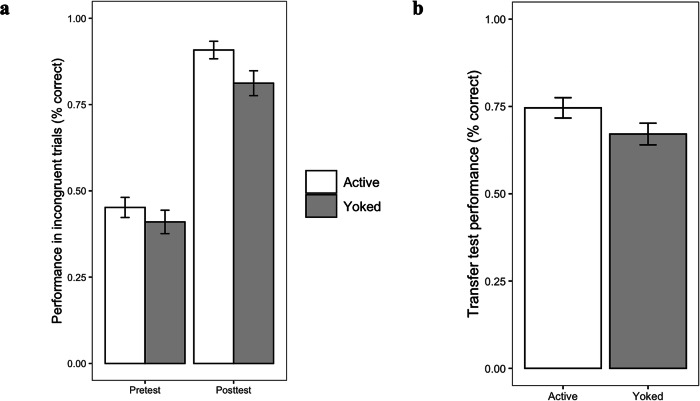
Test performance. **a** Performance improved strongly from pretest to posttest in both groups. While children in the active condition performed significantly better on the posttest than children in the yoked condition, the interaction between condition and time did not reach significance. **b** On the transfer test, children in the active condition performed better than children in the yoked condition. Error bars indicate standard errors.

An overview of children’s performance in the pretest, posttest, and transfer test, separately for the active and yoked condition, is provided in Fig. 1. On the pretest, performance was below chance in both the active (*M* = 0.45, *SD* = 0.20) and yoked condition (*M* = 0.41, *SD* = 0.23). An independent t-test indicated no significant differences between conditions (*t*(93) = 0.96, *p* = 0.337, *d* = 0.20). These data indicate that, as expected, most children had misconceptions about what determines water displacement.

On the posttest, performance of both groups was close to ceiling. Nevertheless, an independent *t*-test indicated that children in the active condition performed significantly better than children in the yoked condition (*t*(93) = 2.21, one-tailed *p* = 0.015, *d* = 0.45; active: *M* = 0.91, *SD* = 0.18; yoked: *M* = 0.81, *SD* = 0.24). A (non-preregistered) repeated measures analysis of variance indicated that children in both conditions significantly improved their performance from pretest to posttest (*F*(1, 93) = 179.77, *p* < 0.001, *d* = 2.78). Additionally, the main effect of condition was significant (*F*(1, 93) = 5.32, *p* = 0.023, *d* = 0.48). However, there was no significant condition by time interaction (*F*(1, 93) = 0.70, *p* = 0.405, *d* = 0.17). These results suggest that the relatively small difference in posttest performance in favor of the active (vs. yoked) condition did not hold when looking at children’s change in performance from pretest to posttest, potentially due to a ceiling effect.

In the transfer test, where performance in both groups was well below ceiling, children in the active condition exhibited significantly better overall performance than children in the yoked condition (*t*(93) = 1.77, one-tailed *p* = 0.040, *d* = 0.36; active: *M* = 0.75, *SD* = 0.20; yoked: *M* = 0.67, *SD* = 0.21). These findings indicate that children in the active condition have internalized the correct concept more deeply than children in the yoked condition. We also explored the different transfer test tasks with separate *t*-tests. Findings suggest that children in the active condition clearly outperformed their counterparts in the yoked condition in the receptive transfer task (*t*(93) = 1.96, one-tailed *p* = 0.027, *d* = 0.40, active: *M* = 4.22, *SD* = 1.43; yoked: *M* = 3.65, *SD* = 1.42). Children in the active condition performed slightly, though not significantly, better than children in the yoked condition on the productive transfer task (*t*(93) = 1.15, one-tailed *p* = 0.127, *d* = 0.24, active: *M* = 9.27, *SD* = 2.61; yoked: *M* = 8.58, *SD* = 3.13) and explicit conceptual task (*χ²*(2) = 3.70, one-tailed *p* = 0.079, Cramer’s *V* = 0.20, active: *M* = 1.42, *SD* = 0.82; yoked: *M* = 1.17, *SD* = 0.95 A repeated measures analysis of variance indicated that there was no significant interaction between transfer test task and condition (*F*(2, 93) = 0.35, *p* = 0.703), however, indicating a similar pattern of results across the three tasks.

### Perceived autonomy is higher in the active condition and predicts learning

Right after the learning phase (i.e., before the posttest), we asked children about their sense of autonomy and motivation while performing the task. Independent *t*-tests indicated that the children in the active condition reported a higher sense of autonomy than the children in the yoked condition (*t*(93) = 2.70, *p* = 0.008, *d* = 0.56, active: *M* = 4.21, *SD* = 0.71, yoked: *M* = 3.71, *SD* = 1.09), but no higher motivation to perform the task (*t*(93) = 0.96, *p* = 0.340, *d* = 0.20, active: *M* = 4.05, *SD* = 0.67, yoked: *M* = 3.92, *SD* = 0.59).

Both children’s sense of autonomy (*r*(93) = 0.23, *p* = 0.028) and their motivation (*r*(93) = 0.26, *p* = 0.012) were significantly related to better posttest performance but not to better transfer test performance (autonomy: *r*(93) = 0.12, *p* = 0.264; motivation: *r*(93) = 0.18, *p* = 0.090). When including autonomy ratings in a model with condition predicting posttest or transfer test performance, the effects of condition are attenuated and non-significant (posttest: *b* = −0.09, *p* = 0.055; transfer test: *b* = −1.48, *p* = 0.100), which is in line with the assumption that perceived autonomy mediates the effects of self-directed activity on learning.

## Discussion

Our findings suggest that actively engaging in the task oneself—doing it yourself (DIY)—supports conceptual learning more effectively than merely observing the actions of others. To test this, we compared children who generated their own predictions before viewing experimental outcomes with children who instead observed the predictions of a fictitious other child. With the help of Bayesian computational modeling, we implemented a novel control condition in which children were yoked to an ideal Bayesian learner that is tuned to their individual prior beliefs. This means that in the yoked condition, the children saw the answers of an “ideal” peer that starts with the same prior beliefs as them. This way, the yoked condition was at least as informative for causal inference as the active prediction condition, meaning that learning success under the two conditions should be comparable from a rational perspective. Children in the yoked condition also did not report lower levels of motivation to learn than children in the active condition, suggesting that the Bayesian Yoking Procedure did not lead to boredom or frustration among participants in the yoking condition due to a mismatch with their own predictions. Since physical activity was held constant as well, we could discern the “pure” effects of DIY. These findings extend prior research reporting benefits of self-directed activity in children^19,32,37,38^ by showing that these benefits can be detected even when children have no control over the interventions or otherwise influence the data they observe.

One explanation for the advantage of making their own predictions over watching a fictitious other child make the same predictions is that children could engage in a more thorough metacognitive evaluation of the competing hypotheses and the relation between the observed results and their hypotheses. In contrast, children who did not have to generate the predictions themselves may have engaged less deeply with the task. This in turn may have led to less elaboration of their causal theories and ultimately shallower theory revision. This interpretation aligns with Sobel and Kushnir’s^20^ explanation for why children benefit from active intervention in causal learning. They proposed that choosing among multiple possible interventions draws attention to the critical relation between actions and outcomes. In their study, children in the yoked condition who passively observed interventions were less able to recognize and learn from those interventions that could differentiate between causal models than children in the active condition. Extending this logic, our findings suggest that even a volitional action with no impact on the observed intervention—such as generating a prediction—can enhance causal learning.

Our findings suggest that the advantage of DIY has to do with the experience of agency. Agency refers to an individual’s capacity to initiate and perform actions, thereby causing changes in the world^39^. More prosaically, it captures the feeling of being in the driver’s seat with regard to our actions^40^. In line with this interpretation, children in the active condition reported feeling more autonomous than children in the yoked condition, and greater feelings of autonomy were associated with better posttest performance. Notably, controlling for autonomy reduced the posttest advantage of the active condition, suggesting that autonomy may partly explain the learning benefits of DIY. Strikingly, the sense of autonomy in the active condition arose from a minimal manipulation—participants had no control over the evidence they observed but merely made a prediction. This modest level of agency may also explain why the correlation with autonomy did not extend to transfer test performance. In fact, the situation created in our experimental paradigm, in which the predictions made were almost identical in both the active and yoked condition, is in some ways similar to “feigned choice” paradigms in which participants are given the illusion of having a choice but this choice likewise does not alter the course of events. Research on feigned choice indicates that participants learn better when thinking they have a choice^7,41^ and perceive this autonomy as inherently rewarding^42^. Our findings underscore these previous findings and add that our minds weigh the act of deliberately doing something ourselves differently from merely observing somebody else do the same thing.

Our findings also speak to the question of whether human causal learning can be understood as the simplest form of rational Bayesian inference^12^. We found that children’s predictions in the active condition closely aligned with those of an optimal Bayesian learner, supporting prior research suggesting that children’s belief revision can be understood as a process of rational Bayesian inference^36,43^. However, from this perspective, it should not make a difference whether learners are making predictions themselves or observing another learner testing their predictions—the informativeness of the evidence is the same. Observing an ideal Bayesian learner that is tuned to a learner’s prior beliefs and performs the prediction–feedback task could even be advantageous, as it emulates an optimal learning path for that learner. Our findings suggest that such models must take into account other factors, such as agentic beliefs, in order to capture the full richness of human learning.

A methodological contribution of our study is that it provides a proof of concept for the feasibility of the Bayesian Yoking Procedure. We chose water displacement as a learning domain because previous research showed that children’s belief revision in this task approximates that of an ideal Bayesian learner^34,36^, thus making it feasible to implement the personalized Bayesian Yoking Procedure. A resulting limitation, however, is that it is currently unclear how well our findings generalize. Future research is needed to confirm that the Bayesian Yoking Procedure is feasible in other learning scenarios as well. Promising candidates beyond physics learning are chemistry, mathematics, and medical reasoning. In addition, future research should examine whether the Bayesian Yoking Procedure offers an advantage over traditional yoking to another person, given the potential for a mismatch between a participant’s own predictions and those of the yoked individual. Finally, it will be important to investigate whether our findings from a fully computerized task extend to hands-on experimentation. Watching a live demonstration performed by another person may engage children in the yoked condition to a greater degree than the computerized setup used in our study.

Another limitation pertains to inconsistent findings regarding the transfer task, with benefits for the active condition in the receptive task but not for the productive or conceptual tasks. One possible explanation is that the receptive transfer task requires less generative processing and is therefore more sensitive to subtle differences in how information is encoded during learning. In contrast, productive and conceptual transfer likely involve higher cognitive demands, including the ability to abstract and reorganize knowledge. It is possible that a minimal manipulation such as ours may not be sufficient to support these more complex forms of transfer. Future research is needed to replicate and understand these findings.

A further limitation is that the optimal Bayesian models we employ here only predict that children will choose the “most certain” responses (i.e., 1, 3, and 5) for both the simulations of the active condition, and the profiles that the yoked condition are assigned to. However, when given the opportunity to choose, children respond with some uncertainty on close to 10% of trials. One reason may be that by-design, our Bayesian models use “optimal” choice algorithms that are biased toward “max-probability rules” (e.g., that we should always pick what is “most likely”). Children, in contrast, may follow a “sampling hypothesis”^44^ and explore less-likely options.

Understanding the role of self-direction is particularly relevant now that machines are increasingly taking over activities from humans, including conducting and even designing experiments. We need to know when activities should better be left in the hands of learners and how we can help learners develop agency over their learning. The current findings suggest that there is a fundamental reason to leave such activities in the hands of learners; doing them themselves helps learners figure out the causal structure of the world.

## Methods

### Preregistration

This study was preregistered on the Open Science Framework (OSF, https://osf.io/2bkzy). We tested preregistered, directed hypotheses using directional analyses. For these analyses, we therefore reported one-tailed *p*-values, which are clearly indicated throughout. Since the focus of this paper is on the Bayesian Yoking Procedure, we decided not to report analyses on the pupillary data (see the second pre-registered hypothesis) here, but to include the data in a separate paper^45^. For transparency, a short report on findings regarding the second pre-registered hypothesis can be found on the OSF (https://osf.io/ktxdv/).

### Participants

The children were recruited and tested in a large science museum in Germany, partly for reasons of convenience and partly because—in our experience—the sample is more heterogeneous in terms of socioeconomic and ethnic background than when recruiting children and parents for studies carried out at our institute. An a priori power analysis using G*Power^46^ was performed that yielded a minimal sample size of *n* = 84. The power calculation was performed with the following settings: Difference between two independent means, effect size *d* = 0.65 (based on pilot results), α = 0.05, ß = 0.90. We tested a total of 138 children to account for possible dropouts and children who already had the correct concept (exclusion rate based on previous studies using a similar paradigm). Based on our pre-registered exclusion criteria, we excluded 15 participants who did not finish the task and 27 children who correctly solved at least 5 out of 6 incongruent trials during the pretest (active: *n* = 15; yoked: *n* = 12), which indicates that they already had the correct concept before our learning task. In addition, one participant was excluded due to data loss. The final sample consisted of 95 children, 46 of whom were girls (48.42%) and 49 were boys (51.57%). Children were randomly assigned to either the explicit (*n* = 49) or yoked condition (*n* = 46; see description of conditions below). The children ranged in age from 5 to 7 years (*M* = 6.04 ± 0.81). Although our research questions did not focus on age-related differences, we confirmed that including age as a covariate in our models did not alter the pattern of results. Prior to the testing, parents provided written informed consent. Additionally, children received a small gift valued at about 5.00 € for their participation. Ethics approval was obtained from the ethics committee at the DIPF | Leibniz Institute for Research and Information in Education.

### Design and stimuli

**Fig. 2:**
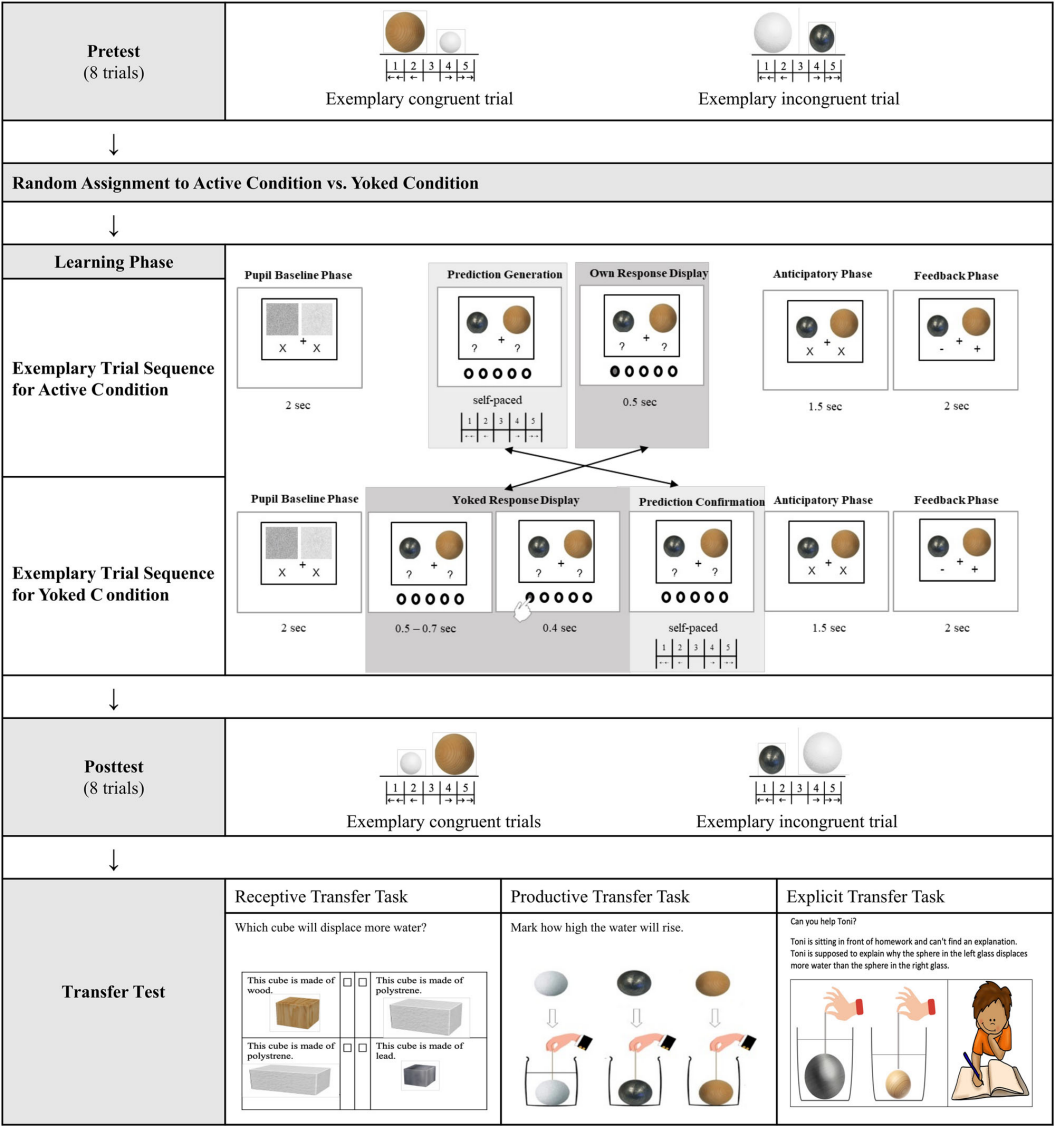
Overview of experimental procedures. The study comprised the following phases: pretest, learning phase, posttest, and transfer test. The figure shows exemplary trials of all phases. The learning phase consisted of 34 computerized trials presented in one of two between-subject conditions: active or yoked. In both conditions, a pupil baseline phase occurred at the beginning of each trial and was only relevant for analyzing the pupil data. In the active condition, children made a prediction about which sphere would displace more water in the *Prediction Generation Phase*. Afterwards, they were shown their prediction again (see *Own Response Display*). After a short *Anticipatory Phase*, they were presented with the correct results in the *Feedback Phase* (“+” indicates that the ball displaces more water, and “–” indicates that the ball displaces less water than the other one; two “+” indicate that both balls displace an equal amount of water). In the yoked condition, children first observed a fictitious child making predictions (see *Yoked Response Display Phase*), then confirmed each prediction in the *Prediction Confirmation Phase*. These fictitious predictions were based on the Bayesian model (see Bayesian Yoking Procedure). After a short *Anticipatory Phase*, they were also presented with the correct results in the *Feedback Phase*.

In the conceptual learning task, children had to learn what factors determine how much water an object displaces. Most children in this age group have the misconception that the material or mass of an object play a role in how much water an object displaces when fully submerged under water^33,34^. To accomplish the task, children thus have to revise their misconception. The experiment consisted of four phases: Pretest, learning phase, posttest, transfer test (see Fig. 2). The stimuli were adapted from a previous study^34^. The pretest, posttest, and learning phase were programmed in and presented with PsychoPy v2022.2.2^47^. The learning phase had two between-subjects conditions: active and yoked. For the learning phase, children were randomly assigned to one of the two conditions. In the active condition, children were tasked with predicting which of two spheres would displace more water. In the yoked condition, children observed the responses of a fictitious other child who had completed the task beforehand. Here, the participating children were required to press the same button as the fictitious child to confirm that they had seen what the fictitious child answered. In both conditions, children saw the correct outcome afterwards.

### Procedure

Children were tested individually in a quiet, adjoining room of the museum. Before the experiment began, the experimenter introduced and demonstrated water displacement by pressing and holding a Styrofoam sphere under water. To ensure that the concept of water displacement was understood, the children were asked to explain what happened to the water when the sphere was held underwater, with the experimenter emphasizing that the sphere was fully submerged. This was done to avoid confusion between water displacement and buoyancy.

The computerized pretest was used to assess children’s conceptual understanding of water displacement. In each trial, the children were presented with pairs of spheres that varied in size (small, medium, large) and material (styrofoam, wood, lead). The pretest consisted of 8 trials, which were designed in such a way as to capture each child’s prior beliefs (see Yoking Procedure below). Six out of the 8 pretest trials were designed to be ‘incongruent’, meaning that the misconception that mass or material would play a role for water displacement would lead to an incorrect answer. For each trial, the children indicated whether they thought the left or the right sphere would displace more water, or whether both spheres would displace the same amount of water. Responses were given on a 5-point scale using a button box (1 = surely the left sphere; 2 = probably the left sphere; 3 = both spheres displace the same amount of water; 4 = probably the right sphere; 5 = surely the right sphere).

For the learning phase, children were randomly assigned to the active or yoked condition and performed a computerized prediction–feedback task. The task was akin to the pretest, but here feedback on the predictions was presented. In addition, eye-movements and pupil dilation were recorded with a camera that was placed below the computer screen and recorded at a frequency of 500 Hz. A fixation cross was presented throughout the learning phase to direct children’s gaze to the center of the screen. Children could only continue with the next trial if they looked at the fixation cross placed in the center of the screen, ensuring that they paid attention to the task.

The learning phase began with 8 practice trials that were constructed in such a way that they could be answered correctly regardless of the child’s prior theory. This was done to familiarize children with the task while minimizing learning. The children then saw 26 learning trials, in 16 of which the mass or material misconception led to an incorrect prediction as the heavier sphere was either smaller or as large as the lighter comparison sphere. All children completed the same trial sequence. Depending on the experimental condition (see Fig. 2), children either made their own predictions before seeing the correct answer (active condition), or observed and confirmed the predictions of a fictitious other child before seeing the correct answer (yoked condition). The fictitious predictions in the yoked condition were generated using a Bayesian model (see Bayesian Yoking Procedure). This procedure ensured that children in the yoked condition were exposed to the same stimuli for the same amount of time as children in the active condition. Additionally, since children in the yoked condition were required to confirm the fictitious child’s responses by pressing a button, their level of physical activity was comparable. Regardless of the predictions generated (or observed), all children saw the correct outcome of each trial. A “+” sign indicates that the ball displaces more water than the other; a “–” sign indicates that the ball displaces less water; and two “+” signs indicate that both balls displace an equal amount of water. The only difference between the two conditions is whether children were asked to make their own prediction or see another “matched” child’s prediction prior to seeing the evidence. Immediately following the learning phase, children completed a short questionnaire assessing their intrinsic motivation and perceived autonomy while performing the learning task (8 items, ω_t_ = 0.71). Two questions related to autonomy (e.g., “I had the say in the tasks.”) and six related to motivation (e.g., “The tasks were fun.”). The autonomy questions were selected to capture children’s experience of agency, as the experience of agency can be understood as the result of actions that are perceived as autonomous^48^. The answers were given on a 5-point Likert scale ranging from “do not agree” to “agree”. The questionnaire can be found on the OSF.

After the learning phase, children completed the posttest (8 trials). The posttest was identical to the pretest, except the position of the spheres (left/right) was reversed.

Next, a paper-pencil transfer test was administered. The transfer test was designed to test whether children could transfer their newly acquired knowledge of water displacement to different tasks, indicating deeper conceptual understanding. The transfer test consisted of a receptive and productive transfer test as well as answering an explicit conceptual question. For the receptive transfer (first six trials), children were asked to determine which of two objects other than spheres (e.g., large plastic crystal vs. medium sized glass crystal; stone vs. beach ball) displaced more water. One point was awarded for each correctly solved trial, for a total of six points.

For the productive transfer, children saw four glasses containing objects of different sizes and materials and were asked to determine how high the water level would rise when the objects were fully submerged in water. Children were presented with two sets, each containing four different objects. In each set, the water level for the first object was already shown and served as a baseline. Children marked how high the water level would rise relative to the baseline water level. The possible pairwise comparisons within each item were scored for accuracy (dummy coded). There were six possible comparisons for each set, for a total of 12 points for productive transfer.

Next, children were tasked with explicitly stating what they thought determined water displacement under the guise of helping a fictional child with doing homework (2 points, conceptual question). We coded whether children correctly argued that it is the size of the object that determines water displacement. Children received 0 points for an incorrect answer, 1 point of partially correct responses that included size (i.e., the size and weight of the object determines water displacement), and 2 points for correct answers. Children could earn a total of 20 points for the transfer test.

After the transfer test, children performed executive functions tasks (i.e., Hearts and Flowers Task, Digit Span Task). These results are not reported here as they were included as a potential moderator to elucidate possible interindividual differences in belief revision, which are not the focus of the current manuscript. The experiment took approximately 35 min to complete.

### Bayesian Yoking Procedure

**Fig. 3:**
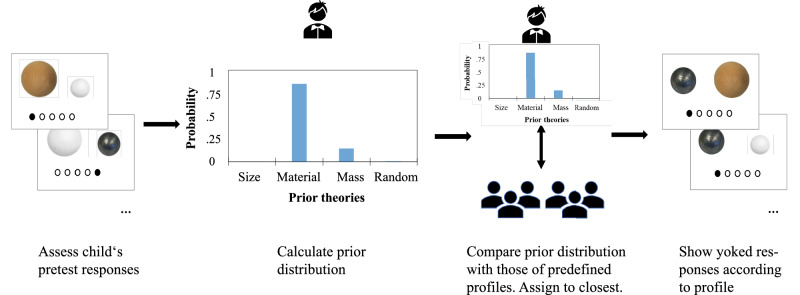
The Bayesian Yoking Procedure. Children are matched with “their” Bayesian learner by (1) using the pretest responses of each child in the yoked condition to approximate individualized prior belief distributions, (2) matching each distribution to one of six predefined profiles and assigning to the best fitting profile such that (3) during the learning phase, children in the yoked condition see the responses associated with their assigned profile.

The Bayesian Yoking Procedure consists of three steps (Fig. 3): (1) creating profiles of the children’s prior belief distributions, (2) determining the corresponding responses during the learning phase for each of the profiles, and (3) assigning new learners to the profiles and present responses accordingly.

First, the profiles were created using the pretest data from a previous study^34^. To determine children’s prior belief models, we considered a hypothesis space distribution of four different, competing theories: a size theory, a material theory, a mass theory, as well as a random guessing model. We included the random guessing model to capture children who may have no intuition about what determines water displacement and thus respond randomly. We simulated the responses that hypothetical participants holding each of the four theories would make on each of the eight pretest trials using a generative probabilistic modeling approach (for details, see ref. ^36^). Given each child’s actual responses during the pretest trials, we could use Bayesian modeling to reverse infer children’s prior models using the distribution of weights on these models for each individual child. This yielded a prior probability distribution of relative weights for each of the four competing theories per individual participant. Then, a k-means algorithm^49^ was used to cluster similar prior distributions across participants into distinct groups. We used the elbow method^50^ to determine the number of clusters, resulting in six clusters. For each of these clusters, the center was determined and selected as the prototypical child.

Second, we determined the responses that a child in the yoked condition observed during the learning phase. To determine the corresponding responses, the prior belief distribution for each of the six prototypical “children” was used to determine how an optimal Bayesian learner that has the same prior theory as that child would respond during the learning phase at that particular trial, given the data they had already observed up until that trial. Thus, starting with the prior probability distributions as revealed in the pretest, we performed trial-by-trial Bayesian posterior updating for each of the 34 trials of the learning phase. We calculated an updated posterior probability at a given trial as in Eq. (1) for each of the four competing theories (*h*_*i*_) after observing the data (*d*), given a prior probability of said theory given the prior data p(*h*_*i*,_*|d*):

1 $$p({h}_{i}|d)=\frac{p(d|{h}_{i})p({h}_{i})}{p\left(d\right)}$$

In other words, the model of each child’s performance was then updated trial-by-trial using Bayes’ rule, starting from the initial priors found in the pretest. This updating took into account both the influence of children’s specific prior theories and the dynamics of active trial-by-trial learning in response to evidence.

Since this procedure was performed for each of the six prototypical children, this resulted in six response profiles—one for each cluster. According to previous findings^36,51^, these response profiles thus reflect the responses that the prototypical child would most likely choose in the 34 learning phase trials. We tested whether our model replicated the previous findings by evaluating whether the responses of children in the active condition aligned with those of an optimal Bayesian learner. Since children in the yoked condition did not make predictions, we could not compute correlations between their responses and the model. Comparing responses in the active condition to the model (ignoring confidence level), we found a strong correspondence: the optimal Bayesian model accurately predicted 80.4% of the responses during the learning phase. To account for the hierarchical nature of the data (responses nested within participants), we ran a linear mixed-effects regression model to examine the relations between the Bayesian model’s predictions and the predictions made by children in the active condition. Results indicated that the Bayesian model was a strong predictor of children’s choices in the active prediction condition (*b* = 0.67, *SE* = 0.017, *z* = 38.91, *p* < 0.001). The random intercept was non-significant (*b* = 0.04; (χ²(1) = 1.52, *p* = 0.217 via Likelihood Ratio Test for random effects), suggesting little heterogeneity in accuracy across participants.

Third, we assigned the new learners we tested for this study to the best fitting of the 6 yoked profiles. The pretest responses of the children in the yoking condition were used to calculate their prior distribution (as was done in step 1), and this distribution was compared to the prior distributions of the six profiles. The children were then assigned to the best matching profile, that is, the profile whose distribution of prior beliefs most closely resembled that of the children (i.e., lowest Wasserstein distance)^52^. During the learning phase, the children saw the responses of the assigned yoked profile. Assignment to matched profiles was for methodological ease as it required pre-coding only 6 different experimental conditions, as opposed to one for each and every child, which would have been required had we matched on an individual basis. A potential concern of assigning to predefined profiles is that perhaps these profiles do not closely align with the actual children, thus leading to children seeing different predictions than if we had used a truly individualized procedure. Inaccuracies could arise due to an insufficient number of clusters or errors in the assignment of participants to the six clusters, which had to be made on the fly during the experiment. To test the accuracy of our Bayesian Yoking Procedure, we tested whether the predictions generated trial-by-trial for our profile-based approach were a reasonable approximation for the observations that an individually matched modeling approach would have made (see “Results”).

## Data Availability

All data, study materials, and the script to run the Bayesian Yoking Procedure are publicly available (https://osf.io/ktxdv/).

## Code availability

Data were analyzed with R (version 4.4.1). All analysis scripts are publicly available (https://osf.io/ktxdv/).
